# Prey preference in a kleptoplastic dinoflagellate is linked to photosynthetic performance

**DOI:** 10.1038/s41396-023-01464-3

**Published:** 2023-06-30

**Authors:** Norico Yamada, Bernard Lepetit, David G. Mann, Brittany N. Sprecher, Jochen M. Buck, Paavo Bergmann, Peter G. Kroth, John J. Bolton, Przemysław Dąbek, Andrzej Witkowski, So-Yeon Kim, Rosa Trobajo

**Affiliations:** 1 Department of Biology, University of Konstanz, Konstanz, Germany; 2 Marine and Continental Waters Program, Institute for Food and Agricultural Research and Technology, La Ràpita, Spain; 3 Royal Botanic Garden Edinburgh, Edinburgh, UK; 4 Electron Microscopy Centre, University of Konstanz, Konstanz, Germany; 5 Department of Biological Sciences, University of Cape Town, Cape Town, South Africa; 6 Institute of Marine and Environmental Sciences, University of Szczecin, Szczecin, Poland; 7 Department of Oceanography, Kunsan National University, Gunsan, Republic of Korea

**Keywords:** Molecular evolution, Cellular microbiology, Photosynthesis

## Abstract

Dinoflagellates of the family Kryptoperidiniaceae, known as “dinotoms”, possess diatom-derived endosymbionts and contain individuals at three successive evolutionary stages: a transiently maintained kleptoplastic stage; a stage containing multiple permanently maintained diatom endosymbionts; and a further permanent stage containing a single diatom endosymbiont. Kleptoplastic dinotoms were discovered only recently, in *Durinskia capensis*; until now it has not been investigated kleptoplastic behavior and the metabolic and genetic integration of host and prey. Here, we show *D. capensis* is able to use various diatom species as kleptoplastids and exhibits different photosynthetic capacities depending on the diatom species. This is in contrast with the prey diatoms in their free-living stage, as there are no differences in their photosynthetic capacities. Complete photosynthesis including both the light reactions and the Calvin cycle remain active only when *D. capensis* feeds on its habitual associate, the “essential” diatom *Nitzschia captiva*. The organelles of another edible diatom, *N. inconspicua*, are preserved intact after ingestion by *D. capensis* and expresses the *psbC* gene of the photosynthetic light reaction, while RuBisCO gene expression is lost. Our results indicate that edible but non-essential, “supplemental” diatoms are used by *D. capensis* for producing ATP and NADPH, but not for carbon fixation. *D. capensis* has established a species-specifically designed metabolic system allowing carbon fixation to be performed only by its essential diatoms. The ability of *D. capensis* to ingest supplemental diatoms as kleptoplastids may be a flexible ecological strategy, to use these diatoms as “emergency supplies” while no essential diatoms are available.

## Introduction

It is well established that plastids in different eukaryote supergroups originated from different algae through multiple endosymbiotic events [1]. According to traditional hypotheses [e.g., [2, 3]], the first step in plastid evolution was engulfment of intact free-living microalgal cells by heterotrophic eukaryotes via phagocytosis, and conversion into a permanent endosymbiont. Other key events occurred after permanent engulfment, including: establishment of metabolite exchange systems between endosymbiont and host, control of endosymbiont cell division by the host, loss of some genes from the endosymbiont, and transfer of other genes (endosymbiotic gene transfer: EGT) from the endosymbiont to the host nucleus.

In contrast, more modern models of plastid evolution—e.g., the “shopping bag model” [4]—suggest that these key events had already begun before the establishment of permanent endosymbionts [4–6], during repeated acquisitions of transiently maintained microalgae known as kleptoplastids [7]. During this evolutionary stage, the kleptoplastic hosts were able to keep ingested microalgal organelles for photosynthesis, but because of the absence of coordinated division of the prey nucleus [8, 9] and/or the deterioration and digestion of prey plastids [10, 11], the hosts constantly needed to feed on new free-living microalgae. Extant kleptoplastic species occur in diverse eukaryotes, e.g., ciliates [12, 13], dinoflagellates [14, 15], foraminifera [16, 17], katablepharids [18], sea slugs [19], and flatworms [20]. EGT from previous plastids or previous endosymbionts, which is suggested by the shopping bag hypothesis, was evidenced in some kleptoplastids or endosymbionts by genomic sequencing [21–23], proteomic analyses [24, 25] and transcriptomic analyses [26–28]. On the other hand, few or no EGT have been found to occur from microalgae that are current sources of the kleptoplastids [26–28]. This is curious, because many kleptoplastic species exhibit strong preference for particular kleptoplastids [14, 18, 29]. We postulate that this reflects species-specific metabolic connections already established between host and prey, before EGT happened.

In this study we investigated whether prey preference is connected with photosynthetic performance in a kleptoplastic dinoflagellate *Durinskia capensis*. This dinoflagellate belongs to the Kryptoperidiniaceae [30], which use diatoms as their plastids [31, 32] and therefore are often called dinotoms. Until *D. capensis* was re-discovered in 2019 [33], all dinotoms had been thought to possess permanent diatom-derived plastids [34]. So far, *D. capensis* is the only dinotom known to be kleptoplastic, although there may be others [35, 36]. Regardless of the evolutionary stage of their plastids, dinotoms maintain almost all diatom organelles [37, 38], except for the diatom plasma membrane and frustule, which are not transferred into the dinotoms during ingestion [33]. The diatom organelles and cytoplasm are separated by a single symbiosome membrane [39] from the dinoflagellate cytoplasm [40]. We refer to the diatom inhabitants of dinotoms as “organelle-retaining diatom-derived plastids” (ODPs), in accordance with symbiont definitions [41, 42].

*D. capensis* feeds on several species of diatoms, some of which prolong the kleptoplastid maintenance period [33]. Only two diatoms, *Nitzschia captiva* [43], which was previously referred to as *N*. cf. *agnita* in [33], and its undescribed very close relative [32], both were found as ODPs by genetic sequencing using natural samples [32, 33], support stable long-term growth of this dinoflagellate in culture. The *D. capensis* + habitual-associate *N. captiva* ± other diatoms system is thus ideal for investigating species-specific metabolic connections between host and preferred prey. In this study, we conducted feeding experiments to determine which diatom species can nourish *D. capensis*, and if this dinoflagellate selects diatoms before ingestion. Based on these results, we selected two diatoms, the habitually associated essential diatom, *N. captiva*, and the edible but non-essential supplemental diatom, *N. inconspicua*, to evaluate photosynthetic parameters and to confirm both ODPs are preserved intact after ingestion by *D. capensis*.

## Results

### Many diatom species positively affect *D. capensis* growth

We first performed a feeding experiment with thirteen diatom species, which were categorized into three groups according to phylogenetic position and source habitat (Table 1, Supplementary Fig. 1): (i) species of the Bacillariaceae collected from the type locality of *D. capensis* (Group 1); (ii) other pennate diatoms collected from this locality (Group 2); and (iii) species of Bacillariaceae collected from other localities (Group 3).

**Table 1:** Diatoms used in this study.

Species name	Strain	Source country	Further information & Accession numbers in NCBI
Group 1: species of Bacillariaceae collected from the type locality of *D. capensis*			
*Hantzschia* cf. *baltica*	IRTA-CC-126	South Africa	LC746289
*Nitzschia captiva*	IRTA-CC-152	South Africa	*Nitzschia* cf. *agnita* in [33], LC482715, LC746294, SAMD00577053, SAMD00577051
*Nitzschia inconspicua*	IRTA-CC-1	South Africa	LC385877
*Psammodictyon* sp.	NY099	South Africa	LC746292
Group 2: Diatoms of other genera collected from the type locality of *D. capensis*			
*Halamphora* sp.	IRTA-CC-2	South Africa	LC746288
cf. *Stauroneis* sp.	IRTA-CC-128	South Africa	LC746291
*Navicula* sp.	IRTA-CC-127	South Africa	LC746290
Group 3: species of Bacillariaceae collected from other places			
*Nitzschia inconspicua*	IRTA-CC-211	Spain	HF675084
*Nitzschia lembiformis*	IRTA-CC-214	Spain	Close relative of ODPs of *D. oculata*, HE802701
*Nitzschia* cf. *pusilla*	CCMP558	Canada	HF675129 & EF423498
*Nitzschia* sp.	SZCZP1124	South Africa	LC385875
*Psammodictyon* sp.	SZCZP1020	South Africa	LC385876
*Simonsenia medliniae*	KNU-Y-19064	South Korea	Close relative of ODPs of *D. kwazulunatalensis*, MW387009

To differentiate the clones of the same species isolated from different places, we added the name of isolated place at the end of the species name.

**Fig. 1:**
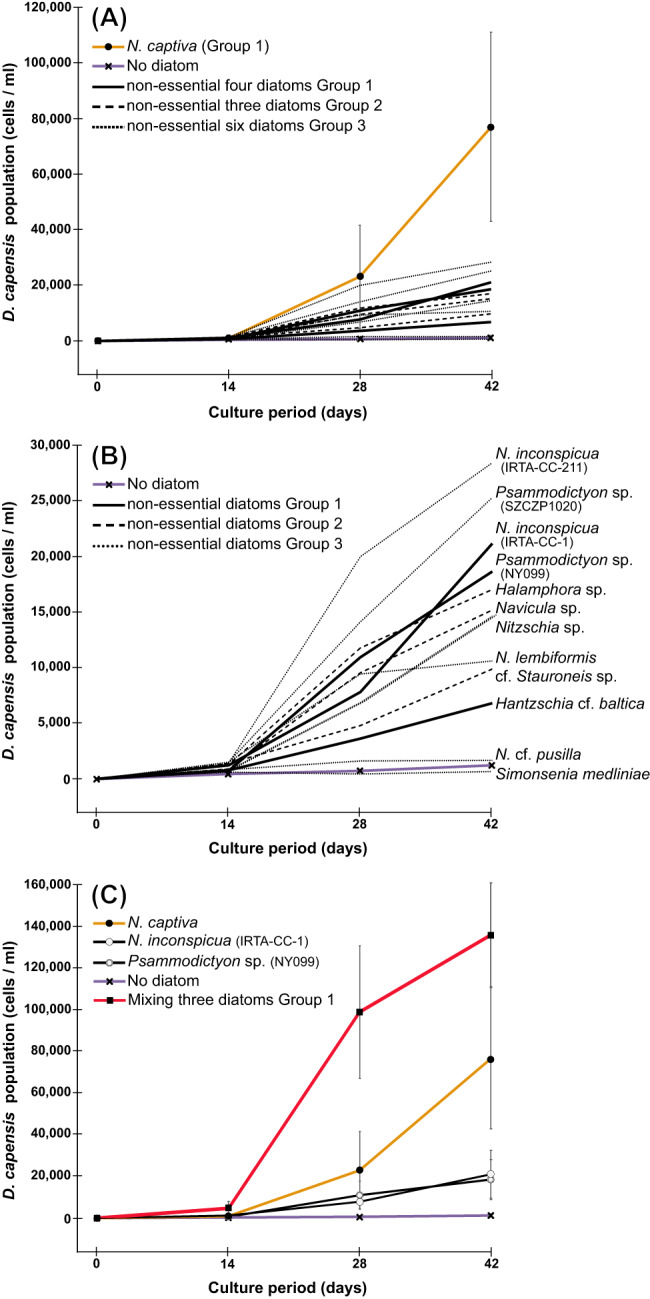
Feeding experiment of *D. capensis* with thirteen diatom species. **A** *D. capensis* co-cultured with thirteen species of diatoms respectively. The essential diatom *N. captiva* was the most influential diatom for *D. capensis* growth; however, the other ten diatoms were also able to increase *D. capensis* numbers, with the exception of two diatoms in Group 3. **B** Detail of (**A**) showing growth of *D. capensis* co-cultured with non-essential diatoms. Except for two diatom species in Group 3, *N*. cf. *pusilla* and *Simonsenia medliniae*, all diatoms used in this study positively influenced *D. capensis* growth. **C** *D. capensis* co-cultured with *N. captiva*, *N. inconspicua* (strain IRTA-CC-1) or *Psammodictyon* sp. (strain NY099), respectively, or co-cultured with these three diatoms together. Co-culturing with three species had the biggest impact on the *D. capensis* growth. Orange line with black circle = *D. capensis* co-cultured with *N. captiva*, Purple line with cross = *D. capensis* mono-cultured in the absence of any free-living diatoms, Black line = *D. capensis* co-cultured with diatoms of Group 1, Broken line = *D. capensis* co-cultured diatoms of Group 2, Dotted line = *D. capensis* co-cultured with diatoms of Group 3, Black line with white circle = *D. capensis* co-cultured with *N. inconspicua* (IRTA-CC-1), Black line with white double circle = *D. capensis* co-cultured with *Psammodictyon* sp. (NY099), Red line with square = *D. capensis* co-cultured with *N. captiva, N. inconspicua* (IRTA-CC−1), and *Psammodictyon* sp. (NY099) together.

Most of the diatoms could support some growth of *D. capensis*, the exceptions being two species of Group 3, *N*. cf. *pusilla* and *Simonsenia medliniae*. The essential diatom *N. captiva* (Group 1) had by far the largest impact on *D. capensis* growth (Fig. 1A), allowing *D. capensis* to increase from an initial 10 cells ml^−1^ to 76,890 cells ml^−1^ on Day 42 (Supplementary Table 1). In the negative control, where no diatoms were added, only 1238 cells ml^−1^ were present on Day 42 (Supplementary Table 1). The other ten diatom species were edible and positively influenced *D. capensis* growth (Fig. 1A and B) but their effects were lower than *N. captiva*. *N. inconspicua* IRTA-CC-211 of Group 3 had the greatest impact of these ten, but only 28,435 cell ml^−1^ were present on Day 42 (Fig. 1B, Supplementary Table 1). There was a synergistic effect when *D. capensis* was co-cultured with *N. captiva* and two supplemental species of Group 1, *N. inconspicua* IRTA-CC-1 and *Psammodictyon* sp. NY099, resulting in 136,357 *D. capensis* cells ml^−1^ on Day 42 (Fig. 1C, Supplementary Table 1), 1.77 times the numbers of cells when only *N. captiva* was co-cultured with *D. capensis*.

### *D. capensis* selectively ingests the essential diatom

The above feeding experiment showed that *D. capensis* was able to feed on a variety of diatom species. To test if *D. capensis* ingests these diatoms selectively, four diatom species were used for a further feeding experiment: *N. captiva*, *N. inconspicua* IRTA-CC-1, which had the second biggest impact on *D. capensis* growth among Group 1 species, *Halamphora* sp., and *Navicula* sp., which had the highest and second highest impacts on *D. capensis* growth in Group 2. The feeding behavior of *D. capensis* changed depending on the diatom species and whether diatoms were present as one cell or in clusters. With *N. captiva*, *D. capensis* started feeding attacks (Supplementary Video 1) within 6 min (Table 2, Supplementary Video 2). With single cell of the three supplemental diatom species, *D. capensis* never started feeding attacks within the 3 h of observation (Table 2, Supplementary Video 3). When adding a clump of supplemental diatoms, *D. capensis* often came close and attacked several times, but then left without feeding (Supplementary Video 4). These observations show that *D. capensis* selects its prey diatoms before feeding, and its first choice is always *N. captiva*.

**Table 2:** Feeding experiment of *D. capensis* with selected four diatom species.

Diatom species	*N. captiva*	*N. inconspicua*	*Halamphora* sp.	*Navicula* sp.
Strain	IRTA-CC-152	IRTA-CC-1	IRTA-CC-2	IRTA-CC-127
Group	1	1	2	2
Relationship with *D. capensis*	Essential	Supplemental	Supplemental	Supplemental
Time to show the feeding behavior (minute:second)	02:12	No feeding attack was observed in 3 h		
	05:54			
	02:47			
	02:51			
	04:24			
Average (minute:second)	03:38	–	–	–

### *D. capensis* contains multiple ODPs in a cell

Two diatoms were selected for further experiments: the essential diatom *N. captiva* and the supplemental diatom showing the second highest impact in Group 1, *N. inconspicua* IRTA-CC-1. We first determined the chlorophyll a (Chl *a*) concentration cell^−1^ prior to measuring their photosynthetic activity, to compare photosynthetic capacity values Chl *a*^−1^. Five samples were prepared: (a) *N. captiva* cultured for a week until the end of exponential phase; (b) *N. inconspicua* cultured similarly; (c) *D. capensis* starved for 3 weeks to eliminate *N. captiva* ODPs and then co-cultured with free-living *N. captiva* for 3 weeks until the end of the dinoflagellate exponential phase; (d) *D. capensis* starved for 3 weeks then co-cultured with *N. captiva* and *N. inconspicua* together for 3 weeks; and (e) *D. capensis* starved for 3 weeks then co-cultured with *N. inconspicua* for 3 weeks. However, due to the high residue of leftover free-living *N. inconspicua* (Supplementary Table 2), sample e was not used in this experiment.

**Fig. 2:**
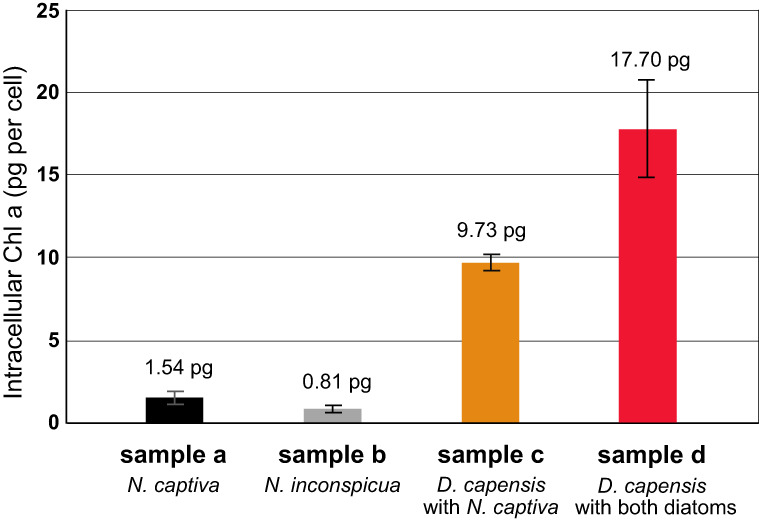
Chl *a* concentration (pg cell^−1^) in the different cultures. Free-living *N. captiva* (sample a; black bar); free-living *N. inconspicua* (sample b, gray bar); *D. capensis* co-cultured with *N. captiva* (sample c, orange bar); and *D. capensis* co-cultured with *N. captiva* and *N. inconspicua* (sample d, red bar).

Results showed that *D. capensis* must keep multiple numbers of ODPs cell^−1^ (Fig. 2). Free-living *N. captiva* (sample a) or *N. inconspicua* (sample b) contained 1.54 pg cell^−1^ ( ± 0.29) or 0.81 pg cell^−1^ ( ± 0.11) of Chl *a*, respectively, while *D. capensis* co-cultured with *N. captiva* (sample c), or with both of them (sample d) contained 9.73 ( ± 0.42) or 17.70 ( ± 3.00) pg of Chl *a* cell^−1^, respectively. The Chl *a* concentration in the dinoflagellate co-cultured with *N. captiva* (sample c) was thus 6.31 times higher than free-living *N. captiva* (sample a). When the dinoflagellate was co-cultured with both diatoms (sample d), the Chl *a* concentration was 1.82 times higher than when *D. capensis* was co-cultured with only *N. captiva* (sample c), indicating that *D. capensis* maintained both diatoms in the cell and preserved more ODPs cell^−1^ when it was co-cultured with the supplemental diatom.

### The essential diatom and a supplemental diatom differ in their photosynthetic capabilities after ingestion

The relative photosynthetic electron transport rate (rETR) was measured by Pulse-Amplitude-Modulation chlorophyll fluorometry (PAM), using the same samples as the Chl *a* measurement. Since the Chl *a* concentration cell^−1^ differed significantly between diatoms and dinotoms (Fig. 2), rETR was measured per 2 μg of Chl *a* rather than per cell.

**Fig. 3:**
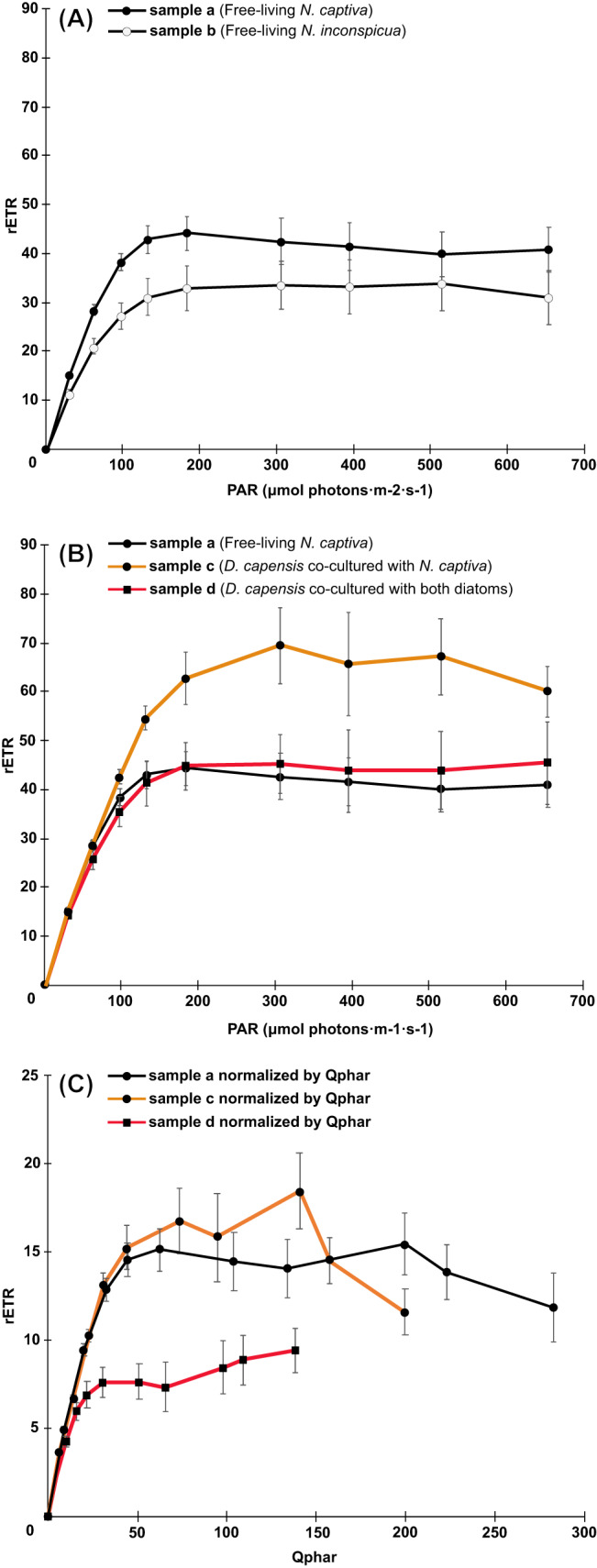
Relative photosynthetic electron transport rates (rETRs) of the free-living and ingested diatoms. **A** rETR values of free-living *N. captiva* (sample a) and free-living *N. inconspicua* (sample b). **B** rETR values of free-living *N. captiva* (sample a), of *D. capensis* co-cultured with *N. captiva* (sample c), and of *D. capensis* co-cultured with *N. captiva* and *N. inconspicua* together (sample d). **C** Q_phar_-normalized rETR values of free-living *N. captiva* before ingestion (sample a), of *D. capensis* co-cultured with *N. captiva* (sample c) and of *D. capensis* co-cultured with *N. captiva* and *N. inconspicua* (sample d). Black line with black circle = sample a, Black line with white circle = sample b, Orange line with black circle = sample c, Red line with square = sample d.

The rETR values of free-living *N. captiva* and *N. inconspicua* were similar (samples a and b: Fig. 3A). After ingestion, ODPs derived from *N. captiva* (sample c) exhibited a higher rETR than free-living *N. captiva* (sample a: Fig. 3B). At first glance, this result seems to indicate that the capacity for the photosynthetic light reaction of *N. captiva* increased after ingestion. However, we reasoned that this was caused by intracellular self-shading of Chl *a*, called the “package effect” [44]: when the amount of Chl *a* maintained in one cell is significantly different, as is the case between *D. capensis* and free-living diatoms (Fig. 2), the cell where the Chl *a* concentration is higher has denser pigment packing and the photosynthetically absorbed radiation (Q_phar_), i.e., the energy absorbed by Chl *a* cell^−1^ driving photosynthesis, becomes smaller. To correct for this, we normalized the rETR of *N. captiva* by the Q_phar_ intensities before and after ingestion. The rETR after normalization by Q_phar_ was the same for the a and c samples (Fig. 3C), indicating that the non-normalized higher rETR value for *N. captiva* after ingestion is due to the package effect, and therefore the photosynthetic performance of *N. captiva* is in fact identical before and after ingestion.

To evaluate the rETR of *N. inconspicua* after ingestion, we measured sample d with *D. capensis* co-cultured with *N. captiva* and *N. inconspicua* (Fig. 3B) and normalized the rETR by Q_phar_ (Fig. 3C). As indicated in Fig. 2. *capensis* in sample d preserved ODPs derived from both *N. captiva* and *N. inconspicua* in the cell. Thus, if both diatoms have similar photosynthetic capacities, the rETRs of samples c and d should be identical. However, the ETR normalized with Q_phar_ dropped strongly in sample d compared to sample c (Fig. 3C), indicating that ODPs derived from *N. inconspicua* are much less efficient in performing photosynthesis than ODPs from *N. captiva*.

### Ingested supplemental diatoms are preserved their organelles intact inside *D. capensis*

**Fig. 4:**
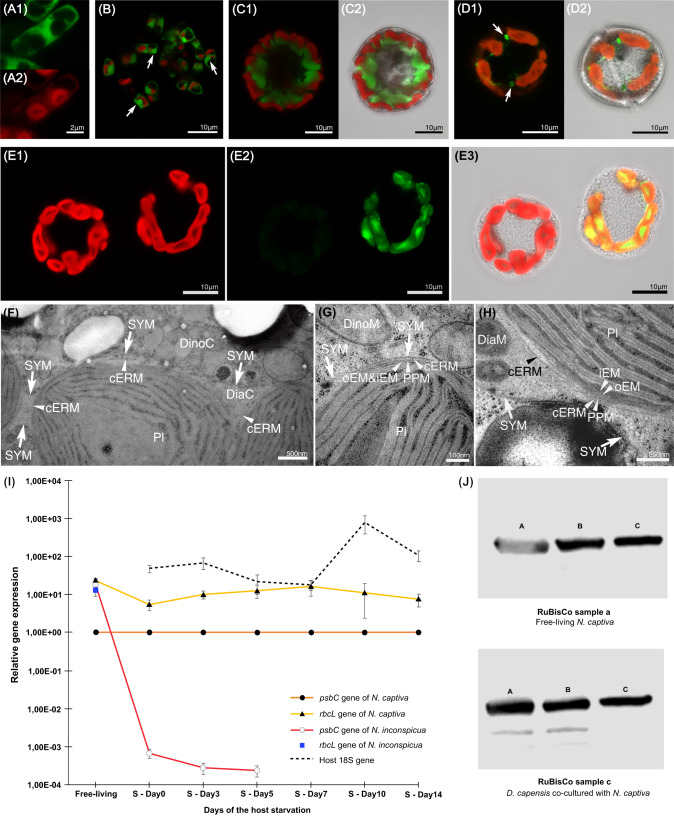
Relative gene expression, rbcL protein expression and organelle retention of diatoms before and after ingestion. **A** Free-living *N. inconspicua* expressing GFP. The GFP was observed in the cytoplasm (A1), but not in the plastids (A2). Green = GFP fluorescence. Red = Chl *a* autofluorescence. **B** Free-living *N. inconspicua* expressing GFP in the nucleus (arrows) and the cytoplasm. **C** *D. capensis* maintained the GFP-expressing cytoplasm of *N. inconspicua*. The cytoplasm was located next to the plastids. C1 = A merged photo of GFP fluorescence and Chl *a* autofluorescence. C2 = A merged photo of GFP fluorescence, Chl *a* autofluorescence and bright filed. **D** *D. capensis* maintained the GFP-expressing two nuclei (arrow) of *N. inconspicua*. The nuclei were also located near to the plastids. D1 = A merged photo of GFP fluorescence and Chl *a* autofluorescence. D2 = A merged photo of GFP fluorescence, Chl *a* autofluorescence and bright filed. **E** Two *D. capensis* cells maintained either of the GFP-expressing *N. captiva* plastids or the wild-type *N. inconspicua* plastids. Plastids derived from *N. captiva* and *N. inconspicua* were impossible to distinguish by their shapes or by their Chl *a* autofluorescence (E1), but became distinguishable by the GFP fluorescence of *N. captiva* plastids (E2 and E3). E1 = Chl *a* autofluorescence photo. E2 = GFP fluorescence photo. E3 = A merged photo of Chl *a* autofluorescence, GFP fluorescence and bright filed. **F**–**H** *N. inconspicua*-derived ODPs inside a *D. capensis* cell. **F** The symbiosome membrane (SYM), which separates diatom cytoplasm (DiaC) and dinoflagellate cytoplasm (DinoC) was visible surrounding the diatom plastids (PI). cERM = the outermost membrane of diatom plastids. **G** and **H** Diatom plastidial membranes were preserved intact inside of the SYM, comprising the innermost membrane (iEM), the second innermost membrane (oEM), the second outermost membrane (PPM), and the cERM. Arrow = SYM, DiaM = Diatom mitochondria, DinoM = Dinoflagellate mitochondria. **I** Relative expression levels of plastid-encoded *psbC* and *rbcL* of *N. captiva* and *N. inconspicua* before (sample a and b) and after ingestion under the starving condition of *D. capensis* (sample d). All gene expressions were normalized with the *psbC* gene of *N. captiva*. Both genes were expressed stably in *N. captiva* before and after ingestion. In *N. inconspicua*; however, only the *psbC* gene expressed after ingestion, although both *psbC* and *rbcL* genes expressed when *N. inconspicua* was free-living. The *psbC* of *N. inconspicua* became undetectable from Day 7 after the starvation (S-Day 7). Orange line with black circle = *psbC* gene of *N. captiva*, Yellow line with triangle = *rbcL* gene of *N. captiva*, Red line with white circle = *psbC* gene of *N. inconspicua*, Blue square = *rbcL* gene of *N. inconspicua*, Broken line = 18 S gene of *D. capensis*. **J** RbcL protein expression levels of *N. captiva* before (sample a) and after ingestion (sample c). *N. captiva* produced rbcL protein in both samples. A = 1 μg of Chl *a* of sample volume; B = 0.5 μg of Chl *a* of sample volume; C = 0.25 μg of Chl *a* of sample volume.

To investigate whether ingested *N. inconspicua*-derived ODPs are preserved intact by *D. capensis*, we fed *D. capensis* either 1) with *N. inconspicua* expressing the green fluorescent protein (GFP) in the cytoplasm (Fig. 4A) and nucleus (Fig. 4B), or 2) with wild-type *N. inconspicua* and *N. captiva* expressing GFP in the plastid. Confocal laser scanning microscopy (CLSM) confirmed that *D. capensis* preserved the GFP-expressing cytoplasm (Fig. 4C) and nuclei (Fig. 4D) of *N. inconspicua*. Both wild-type *N. inconspicua* plastids and GFP-expressing *N. captiva* plastids were maintained intact, spread widely over the inner surface of *D. capensis* cells (Fig. 4E and Supplementary Fig. 2). Further observations by transmission electron microscopy (TEM) confirmed that ingested diatom plastids were preserved intact regardless of diatom species, as in the species description study of *D. capensis* [38] and in permanently maintained ODPs of other dinotoms [39, 40, 45]. In *N. captiva-*derived ODPs, the outermost membrane ( = chloroplast endoplasmic reticulum membrane, cERM) and the second outermost membrane ( = periplastid membrane, PPM) were clearly observed (Supplementary Fig. 3A and B). The cERM was connected with the diatom nuclear envelope (Supplementary Fig. 3C–E), suggesting that other plastidial membranes also remained intact after ingestion. In *N. inconspic*ua-derived ODPs, we observed four plastidial membranes: the cERM, the PPM, the second innermost membrane ( = outer envelope membrane; oEM), and the innermost membrane ( = inner envelope membrane; iEM). All these membranes were maintained inside the symbiosome membrane (Fig. 4F–H).

### The supplemental diatom loses *rbcL* gene expression after ingestion

To see whether *N. captiva*-derived ODPs had the ability to perform full photosynthesis from light reaction to carbon fixation, and whether ingested *N. inconspicua* performed any part of photosynthesis, RT-qPCR was used to examine the transcription of photosynthetic-related genes for *N. captiva* and *N. inconspicua* before and after ingestion. Two photosynthetic-related genes were selected: *psbC*, which encodes one of the photosystem II (PSII) inner antenna proteins, and *rbcL*, which encodes the large subunit of RuBisCO, a key enzyme component in the Calvin-Benson-Bassham (CBB) cycle. The *psbC* and *rbcL* were stably expressed in *N. captiva* both before and after ingestion, and this continued until Day 14 after ingestion during the *D. capensis* starved condition (S-Day 14) (Fig. 4I). In contrast, *N. inconspicua* expressed only *psbC* after ingestion (Fig. 4I) and the expression level was already significantly lower on Day 0 and completely undetectable from S-Day 7 (Fig. 4I). We confirmed that the expressed *rbcL* gene of *N. captiva*-derived ODPs was translated into rbcL protein via a western blot (Fig. 4J).

## Discussion

### *D. capensis* feeds on many diatom species but the effects differ

Our results show that most of the diatom species tested positively affect *D. capensis* growth. The most influential diatom is the essential diatom *N. captiva*. Other diatoms are edible but have considerably less effect on *D. capensis* growth, up to a maximum of one-half of the impact of *N. captiva*. However, although *N. captiva* is the most influential diatom for *D. capensis*, the highest growth of this dinotom is found when it is co-cultured with *N. captiva* and other supplemental diatoms together. This kleptoplastic dinotom most likely feeds on several diatom species simultaneously in the natural habitat. Our feeding experiments indicate that *D. capensis* recognizes its essential diatom before feeding, probably by species-specific chemical interactions, and *N. captiva* is fed upon preferentially. Due to this prey selection before feeding, the ingestion mechanisms for supplemental diatoms were not observed, probably because this is a relatively rare event that will be detected only after very long-starved cultures of *D. capensis*: At present it remains open whether supplemental diatoms are also fed upon via myzocytosis, as with *N. captiva* [33].

The impact of different diatom species on *D. capensis* growth is not strongly correlated with source locality and phylogenetic position. The top five species influencing growth of *D. capensis* comprise three species isolated from its type locality, Kommetjie in South Africa, one from another South African locality and one from Spain. However, one of the other diatoms isolated from Kommetjie, *Hantzschia* cf. *baltica*, does not contribute strongly to *D. capensis* growth. The five most influential diatoms belong to the family Bacillariaceae, but they belong to different clades (Supplementary Fig. 1), which have been suggested to represent different genera [46]. We hypothesize that *D. capensis* selects its essential diatoms for other reasons, such as the ease with which the diatom cells can be taken up into *Durinskia*.

### *D. capensis* might use supplemental diatoms for supply of ATP and NADPH

Our data reveal that *D. capensis* maintains the plastids, cytoplasm and nucleus of the supplemental diatom *N. inconspicua*, and arranges them similarly to *N. captiva*. This contradicts the normal fate of prey microalgae ingested by heterotrophic or mixotrophic protists, in which no such orderly spatial arrangement occurs and prey Chl *a* is generally degraded within a few hours to avoid the generation of reactive oxygen species [47]. This, together with the albeit limited continuing expression of a PSII antenna protein gene (*psbC*), is a strong indicator that *N. inconspicua* is maintained by *D. capensis* for performing photosynthesis. However, the photosynthetic activity of *N. inconspicua* decreases significantly after becoming an ODP and the gene encoding for the key enzyme of the CBB cycle, RuBisCO, loses transcriptional activity. We suggest, therefore, that the CBB cycle is completely inactive and that it is only the photosystems that are functional in supplemental ODPs.

**Fig. 5:**
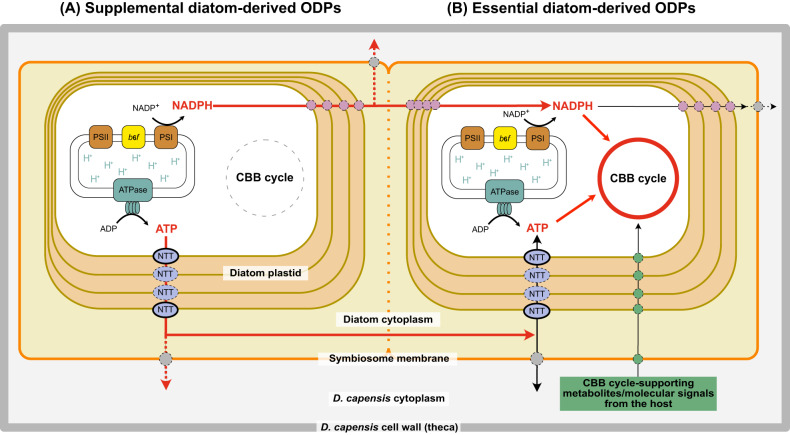
Diagram of ATP and NADPH usages in *D. capensis* containing supplemental and essential diatoms. We hypothesize that ODPs from essential diatom and those from supplemental diatom are kept in the same symbiosome compartment in a single *D. capensis* cell, based on an observation that ODPs derived from different diatom species can be preserved at the same time in a single *D. capensis* cell (Supplementary Fig. 2). This is indicated by a broken symbiosome membrane. **A** Supplemental diatom ODPs. The linear electron flow in the photosystems still works, while the CBB cycle loses activity. ATP and NADPH produced in the supplemental ODPs are therefore transported into the diatom cytoplasm via ATP and NADPH transporters/transporting systems on the diatom four plastidial membranes. After then, they are further transported into the *D. capensis* cytoplasm (red broken line) via unknown transporters on the symbiosome membrane, or into the CBB cycle of essential diatom-derived plastids (red smooth line) via ATP and NADPH transporters on the four plastidial membranes of essential diatoms. Two of the ATP transporters on the plastidial membranes (NTT: nucleotide translocators; with black smooth line) so far have been identified [49, 50] in free-living diatoms. The transporters indicated with the dotted lines have not yet been identified. **B** Essential diatom ODPs. So far, only two diatoms, *N. captiva* and its undescribed close relative have been found as essential diatoms of *D. capensis*. Both the photosystems and the CBB cycle are ideally functional as with free-living essential diatoms, because of the result of species-specific metabolic support from *D. capensis* (green circle).

Expression of *psbC* gene in *N. inconspicua* and the rETR rates, based on PSII fluorescence, suggest linear electron flow is being used to drive ATP and NADPH synthesis, rather than cyclic flow occurring between PSI and cytochromes [48]. The functions of the ATP and NADPH could be (1) to transport into the dinoflagellate cytoplasm for consumption; or (2) to help drive the active CBB cycle of ODPs derived from the essential diatoms (Fig. 5). In both cases, the produced ATP and NADPH would first need to be transported across the four plastidial membranes into the diatom cytoplasm. Two ATP transporters have been identified, in the iEM [49] and in the cERM [50] (Fig. 5A). There are no reports identifying NADPH transporters in diatoms; however, it is known that NADPH produced in diatom plastids is transported to mitochondria in order to balance the plastidial ATP/NADPH ratio for optimal photosynthetic performance [51]. This suggests there is also a NADPH transporting system from diatom plastids to diatom mitochondria across the four plastidial membranes and two mitochondrial membranes (Fig. 5A), most likely by a malate shuttle. For export of ATP and NADPH from the diatom cytoplasm into the dinoflagellate, there needs to be transport across the symbiosome membrane (Fig. 5A). So far, no symbiosome transporters for ATP and NADPH have been identified; however, there is evidence that appropriate metabolic connections may exist between ODPs and the host dinoflagellate [39, 52].

Such partially functional kleptoplastids are also known in an undescribed kleptoplastic dinoflagellate of the Kareniaceae [29], which feeds specifically on the haptophyte *Phaeocystis antarctica*, and in kleptoplastic sea slugs, which feed on various green algae [19]. In the Kareniaceae–*Phaeocystis* system, comparative transcriptomics of free-living *P. antarctica* and *P. antarctica*-derived kleptoplastids have revealed that PSII components are not transcribed in the kleptoplastids, and it is suggested that only cyclic electron transport [48] is active [28]. Since transcription of genes (*petF* and *petH*) associated with NADPH production is also lost in ingested *P. antarctica*, most likely *P. antarctica*-derived kleptoplastids loses CBB cycle activity, like *N. inconspicua*. In more than 50 species of kleptoplastic sea slugs that have been studied, only six species of green algae perform full photosynthesis including carbon fixation [19]. In others, some alga-derived kleptoplastids keep photosystem activity but lose carbon fixation capacity within a few days, while some lose all photosynthetic activity and are preserved by the hosts as slowly digestible food reserves [53].

Supplemental diatoms may also be fed upon for non-photosynthetic purposes in addition to the photosynthetic function: for example, non-photosynthetic plastids of osmotrophic colorless *Nitzschia* spp. have lost all genes for photosynthesis [54], but some plastidial biosynthesis pathways, such as amino acid synthesis or pentose phosphate pathway are retained [55]. Such non-photosynthetic functions have been also identified in kleptoplastic ciliate *Mesodinium rubrum* [56], whose kleptoplastids synthesize amino acids and vitamins for their hosts. In fact, the host dinoflagellates of dinotoms themselves maintain their original red-alga-derived plastids as a non-photosynthetic eyespot [57]. Although it has lost its photosynthetic function, it retains metabolic activity for the heme and methylerythritol phosphate/1-deoxy-D-xylulose-5-phosphate (MEP/DOXP) pathways [58]. Hence the possibility of a non-photosynthetic role for ODPs derived from supplemental diatoms in *D. capensis* seems realistic.

It is known that each dinotom species with permanently maintained ODPs utilizes a specific diatom species that differ from the ODPs of other dinotom species [32]. Our study reveals the flexibility with which a kleptoplastic dinotom is able to use supplemental diatoms for incomplete photosynthesis, and this may drive the species variety of ODPs in dinotoms. This flexibility is also useful in circumstances where *D. capensis* is unable to find its essential diatoms, which may happen quite often: since 2014 we had searched for the essential diatoms, but only once found a free-living cell of *N. captiva* in a 2018 sample. Furthermore, in our early spring to summer meta-barcoding and microscopic observations of habitat samples collected at the *D. capensis* type locality, the essential diatoms were found only as ODPs of *D. capensis* (Trobajo et al. in preparation). Free-living essential diatoms most likely become rare during the active season of *D. capensis* due to strong feeding selection and this dinoflagellate therefore needs emergency supplements to survive.

### Establishing a CBB cycle support system is the first step to convert ingested microalgae into fully functional kleptoplastids

Unlike ODPs from supplemental diatoms, those from the essential diatom *N. captiva* can carry out full photosynthesis, including both the light reactions and the CBB cycle. This allows *D. capensis* to grow photoautotrophically and indicates that *N. captiva* is already metabolically connected with *D. capensis* and is selected as its future permanent ODP. From our survey of co-occurring diatom species, the relationship between *D. capensis* and *N. captiva* is species-specific. *D. capensis* has established metabolic supporting systems for the CBB cycle that only function in relation to the essential diatom (Fig. 5B). This might be a very early but crucial evolutionary step for transforming ingested microalgae into kleptoplastids performing complete photosynthesis.

## Materials and methods

### Sample collection and culture

Natural samples were collected at the type locality of *D. capensis* [38] in September and October 2018 for diatoms and February 2019 for the dinoflagellate. A clonal culture of *D. capensis* (strain NY101) was established using IMK medium (Nihon Pharmaceutical Co., Japan), while six diatoms (Table 1) were established in f/2-Si medium [59]. Strain NY101 can be maintained permanently only by co-culturing with its essential diatom, *Nitzschia captiva* (strain IRTA-CC-152), which was previously called *Nitzschia* cf. *agnita* [33]. Other diatoms used in this study were commercial or private strains from other projects (Table 1). Strains were cultured at 20 °C with 50 µmol photons m^−2^ s^−1^ light under a 16:8 h light:dark cycle. They were identified by 18 S gene sequence and morphology (*D. capensis*) or by frustule morphology and a molecular phylogeny inferred from *rbcL* sequences (diatoms, Supplementary Fig. 1).

### Feeding experiments

Thirteen diatom species (Table 1) were used. Fifty cells of *D. capensis*, which had been starved for 3 weeks to eliminate previously ingested *N. captiva* ODPs, and 15 cells of each diatom species were co-cultured in 5 ml of IMK medium. Negative and positive controls were: (i) a culture containing only 50 cells of *D. capensis* and; (ii) one containing 50 *D. capensis* and 15 cells of three Group 1 diatoms (5 cells *N. captiva*, 5 cells *N. inconspicua* strain IRTA-CC-1, 5 cells *Psammodictyon* sp. strain NY099). After 2, 4, and 6 weeks, 10% of the total sample volume was transferred to a Sedgewick–Rafter Chamber (VWR, Germany) and fixed in Lugol’s solution for cell counts. Four series of experiments were carried out for each culture.

For a second feeding experiment, starved *D. capensis* were prepared, again by culturing for 3 weeks in the absence of free-living diatoms. Single cells or cell clumps of four selected diatom species: *N. captiva* and *N. inconspicua* in Group 1, and *Halamphora* sp. and *Navicula* sp. in Group 2, were isolated under the microscope and transferred to cultures of starved *D. capensis*. The mixed cultures were observed for 3 h or until *D. capensis* showed feeding attacks (Supplementary Video 1).

### Determination of Chl *a* concentration before and after ingestion

Five samples were prepared: (a) *N. captiva* cultured for a week until the end of exponential phase; (b) *N. inconspicua* cultured similarly; (c) *D. capensis* starved for 3 weeks, then co-cultured with *N. captiva* for 3 weeks until the end of the dinoflagellate exponential phase; (d) *D. capensis* starved for 3 weeks, then co-cultured with both diatoms for 3 weeks; and (e) *D. capensis* starved for 3 weeks, then co-cultured with *N. inconspicua* for 3 weeks. The experiments were done in triplicate for each sample.

Free-living diatoms (samples a and b): Chl *a* concentration ml^−1^ was determined by spectrophotometry (Ultrospec 2100 pro, VWR, Germany) according to [60]. Chl *a* cell^−1^ was calculated by counting the number of cells ml^−1^ for each sample using a Sedgewick–Rafter Chamber (VWR, Germany).

ODPs of *D. capensis* (samples c–e): *D. capensis* culture was filtered through 50 μm plankton net to remove leftover clumps of free-living diatoms; counts of free-living diatoms comprised of <10% of total (dinoflagellate + diatom) cell numbers (Supplementary Table 2). Due to the high residual of free-living *N. inconspicua* in sample e (Supplementary Table 2), this sample was not used in this experiment. Chl *a* ml^−1^ of the filtered sample was measured based on [60].

### Fluorescence analysis of diatoms before and after ingestion

Since Chl *a* concentrations cell^−1^ were significantly different between diatoms and *D. capensis* (Fig. 2), cell amounts equivalent to 2 μg of Chl *a* sample^−1^ were used in an IMAGING-PAM (Heinz Waltz, Germany) and rapid light curves were recorded with increasing blue light intensities. rETRs were calculated from *Fs* and *Fm’* values based on [61].

### Q_phar_ of diatoms before and after ingestion

Three samples, a, b and d, were prepared as described in “Determination of Chl *a* concentration before and after ingestion”. After confirming that contamination by leftover free-living diatoms was <10% (Supplementary Table 2), we determined Chl *a* spectrophotometrically, based on [60]. Each sample was used to determine the absorption spectrum of Chl *a* at each wavelength between 400 and 700 nm. This was then used to calculate Q_phar_ based on [44]. The calculated Q_phar_ was used to normalize the pre-calculated rETR, based on [61]. The experiment was done in triplicate for each sample.

### Plastidial transformation of *N. captiva* and *N. inconspicua*

*N. captiva* expressing GFP in the plastids was created in [62], while *N. inconspicua* expressing GFP in nucleus and the cytoplasm was created in this study. *N. inconspicua* was transformed using the GFP-expression vector pCfuNca-Plastid [62]. The *N. inconspicua* transformants expressed GFP in the cytoplasm and the nucleus, even though the plastid contained the diatom-specific plastidial signal and transit peptides, including an ASAFAP motif [63, 64], designed to target diatom plastids. The vector map, vector sequence, methods of transformation and confirmation of genetic transformation (Supplementary Fig. 4) were as in [62]. We prepared *D. capensis* co-cultured with GFP-expressing *N. inconspicua*, and *D. capensis* co-cultured with GFP-expressing *N. captiva* and wild-type *N. inconspicua*. To remove previously ingested *N. captiva* from *D. capensis*, we starved *D. capensis* for 9 weeks, then co-cultured the starved dinoflagellate with diatoms for a further 3 weeks. Based on previous observations [33], ingested *N. captiva* is digested within 12 weeks and the non-GFP expressing plastids of sample d were therefore derived from wild-type *N. inconspicua*.

### Transmission electron microscopy

Two samples, c and e, were prepared as described in “Determination of Chl *a* concentration before and after ingestion”, but we used *D. capensis* starved for 9 weeks, to remove previously ingested *N. captiva* from *D. capensis*. The protocol for transmission electron microscopy was published previously in [39].

### Designing primers for RT-qPCR

The *psbC* sequences of *N. captiva* or *N. inconspicua* (accessions LC746293 and LC746294) were acquired using diatom specific primers psbC22 + psbC1154 [65] (Supplementary Table 3), while their *rbcL* genes (LC482715 and LC385877) were sequenced previously [32, 33]. The *psbC* and *rbcL* gene sequences of *N. captiva* were then used to design RT-qPCR primers on Primer 3 (https://primer3.ut.ee). Candidate primers were confirmed for species-selectivity by comparing with those of *N. inconspicua* on MEGA X [66] and triple-checked by PCR and by melting curves via RT-qPCR (Supplementary Fig. 5). We confirmed that these primers amplified the target genes of the target diatoms by sequencing RT-qPCR amplicons of Day 0 (Supplementary Table 3).

### Sample preparation, RNA extraction, cDNA synthesis and RT-qPCR of diatoms before and after ingestion

To reduce the variation of gene expression level with time, all samples were harvested between 12:00 and 12:30.

Free-living diatoms: samples a and b were prepared as in “Determination of Chl *a* concentration per cell before and after ingestion”. RNA was extracted using a combination of peqGold RNA pure and peqGold total RNA kits (VWR, Germany), purified to remove gDNA contamination by peqGold DNase Digest kit, then used as template for cDNA synthesis with PrimeScript RT reagent kit with gDNA Eraser (Takara Bio Europa, France). Synthesized cDNAs were used as templates for RT-qPCR (GoTaq qPCR and RT-qPCR systems, Promega GmbH, Germany). Cycle threshold values and gene amplification efficiencies were determined with Real-Time PCR Miner 4.0 (http://ewindup.info/miner/). Relative transcript levels were calculated according to [67]. The experiment was done in triplicate for each sample.

ODPs of *D. capensis*: sample d was prepared as described in “Determination of Chl *a* concentration per cell before and after ingestion”. This *D. capensis* sample was then filtered through 50 μm plankton net to remove leftover diatom clumps and the contamination rate of free-living diatoms was checked with a Sedgewick–Rafter chamber (Supplementary Table 2). The filtered *D. capensis* was fixed with liquid nitrogen (Day 0) or re-cultured in fresh IMK medium for 3, 5, 7, 10, and 14 days in the absence of free-living diatoms. To prevent the increase of bacteria and leftover free-living diatoms, 50 μg ml^−1^ penicillin, 50 μg ml^−1^ kanamycin, and 1 μg ml^−1^ GeO_2_ were added to these re-cultured samples. Each sampling day, cultures were checked for leftover free-living diatoms (Supplementary Table 2), fixed with liquid nitrogen, and kept in −80 °C until RNA extraction. RNA extraction, cDNA synthesis, RT-qPCR, and data analysis were performed in the same way as for free-living diatoms. Three series of experiments were set up for each sample.

### Western blot

To reduce time-related variation in protein expression, we harvested all samples between 12:00 and 12:30. Samples a and c were prepared as described in the section “Determination of Chl *a* concentration per cell before and after ingestion”. Protein extraction and separation followed [68] but used 14% lithium dodecyl sulfate-polyacrylamide gel electrophoresis for the protein separation. Samples corresponding to 1 μg, 0.5 μg, or 0.25 μg of Chl *a* were loaded on the gel. Proteins were blotted on an Amersham Protran nitrocellulose membrane (GE Healthcare, GBR) using the semidry blotting technique by means of a Biorad Trans-Blot Turbo system (Hercules, USA). Anti-*rbcL* (AS03 037, Agrisera, Sweden, 1:10,000 dilution) was used for antibody detection. After binding of secondary antibody for 1 h (goat anti-rabbit IgG, HRP conjugated, AS09 602, Agrisera, Sweden; 1:20,000 for *rbcL*), signals were detected using Roti-Lumin Plus (Carl Roth, Germany) in an Odyssey FC Imaging System (LI-COR, USA).

## Supplementary information

Supplementary figures and tables

Supplementary Video 1

Supplementary Video 2

Supplementary Video 3

Supplementary Video 4

## Data Availability

Newly acquired sequences of used diatoms are deposited in the NCBI (LC746288-LC746294). The vector map, vector sequence, methods of transformation of *N. captiva* and *N. inconspicua* are available in [62].

## References

[1] Archibald JM (2015). Endosymbiosis and eukaryotic cell evolution. Curr Biol.

[2] Sagan (Margulis) L (1967). On the origin of mitosing cells. J Theoretic Biol.

[3] Cavalier-Smith T, Lee JJ (1985). Protozoa as hosts for endosymbiosis and the conversion of symbionts into organelles. J Protozool.

[4] Larkum AWD, Lockhart PJ, Howe CJ (2007). Shopping for plastids. Trends Plant Sci.

[5] Keeling PJ (2013). The number, speed, and impact of plastid endosymbiosis in eukaryotic evolution. Annu Rev Plant Biol.

[6] Bodył A (2018). Did some red alga-derived plastids evolve *via* kleptoplastidy? A hypothesis. Biol Rev.

[7] Schnepf E, Elbrächter M (1988). Cryptophycean-like double membrane-bound chloroplast in the dinoflagellate, *Dinophysis* Ehrenb.: Evolutionary, phylogenetic and toxicological implications. Botan Acta.

[8] Onuma R, Horiguchi T (2015). Kleptochloroplast enlargement, karyoklepty and the distribution of the cryptomonad nucleus in *Nusuttodinium* (=*Gymnodinium*) *aeruginosum* (Dinophyceae). Protist.

[9] Kim M, Drumm K, Daugbjerg N, Hansen PJ. Dynamics of sequestered cryptophyte nuclei in *Mesodinium ruburum* during starvation and refeeding. Front Microbiol. 2017; 10.3389/fmicb.2017.00423. 10.3389/fmicb.2017.00423 PMC5359308 28377747

[10] Park MG, Park JS, Kim M, Yih W (2008). Plastid dynamics during survival of *Dinophysis caudata* without its ciliate prey. J Phycol.

[11] Kim M, Kim KY, Nam SW, Shin W, Yih W, Park MG (2014). The effect of starvation on plastid number and photosynthetic performance in the kleptoplastidic dinoflagellate *Amylax triacantha*. J Eukaryot Microbiol.

[12] Johnson MD, Oldach D, Delwiche CF, Stoecker DK (2007). Retention of transcriptionally active cryptophyte nuclei by the ciliate *Myrionecta rubra*. Nature.

[13] Garcia-Cuetos L, Moestrup Ø, Hansen PJ (2012). Studies on the genus *Mesodinium* II. Ultrastructural and molecular investigations of five marine species help clarifying the taxonomy. J Eukaryot Microbiol.

[14] Park MG, Kim S, Kim HS, Myung G, Kang YG, Yih W (2006). First successful culture of the marine dinoflagellate *Dinophysis acuminata*. Aquat Micro Ecol.

[15] Takano Y, Yamaguchi H, Inouye I, Moestrup Ø, Horiguchi T (2014). Phylogeny of five species of *Nusuttodinium* gen. nov. (Dinophyceae), a genus of unarmoured kleptoplastidic dinoflagellates. Protist.

[16] Bernhard JM, Bowser SS (1999). Benthic foraminifera of dysoxic sediments: Chloroplast sequestration and functional morphology. Earth-Sci Rev.

[17] Schmidt C, Morard R, Romero O, Kucera M. Diverse internal symbiont community in the endosymbiotic foraminifera *Pararotalia calcariformata*: Implications for symbiont shuffling under thermal stress. Front Microbiol. 2018; 10.3389/fmicb.2018.02018. 10.3389/fmicb.2018.02018 PMC6141668 30254612

[18] Okamoto N, Inouye I (2006). *Hatena arenicola* gen. et sp. nov., a katablepharid undergoing probable plastid acquisition. Protist.

[19] de Vries J, Christa G, Gould SB (2014). Plastid survival in the cytosol of animal cells. Trends Plant Sci.

[20] van Steenkiste NWL, Stephenson I, Herranz M, Husnik F, Keeling PJ, Leander BS. A new case of kleptoplasty in animals: Marine flatworms steal functional plastids from diatoms. Sci Adv. 2019; 10.1126/sciadv.aaw4337. 10.1126/sciadv.aaw4337 PMC6636991 31328166

[21] Bhattacharya D, Price DC, Yoon HS, Yang EC, Poulton NJ, Andersen RA, et al. Single cell genome analysis supports a link between phagotrophy and primary plastid endosymbiosis. Sci Rep. 2012; 10.1038/srep00356. 10.1038/srep00356 PMC3322482 22493757

[22] Curtis BA, Tanifuji G, Burki F, Gruber A, Irimia M, Maruyama S (2012). Algal genomes reveal evolutionary mosaicism and the fate of nucleomorphs. Nature.

[23] Nowack ECM, Price DC, Bhattacharya D, Singer A, Melkonian M, Grossman AR (2016). Gene transfers from diverse bacteria compensate for reductive genome evolution in the chromatophore of *Paulinella chromatophora*. Proc Natl Acad Sci USA.

[24] Frommolt R, Werner S, Paulsen H, Goss R, Wilhelm C, Zauner S (2008). Ancient recruitment by chromists of green algal genes encoding enzymes for carotenoid biosynthesis. Mol Biol Evol.

[25] Dorrell RG, Gile G, McCallum G, Méheust R, Bapteste EP, Klinger CM, et al. Chimeric origins of ochrophytes and haptophytes revealed through an ancient plastid proteome. eLife 2017; 10.7554/eLife.23717. 10.7554/eLife.23717 PMC5462543 28498102

[26] Wisecaver JH, Hackett JD. Transcriptome analysis reveals nuclear-encoded proteins for the maintenance of temporary plastids in the dinoflagellate *Dinophysis acuminata*. BMC Genom. 2010; 10.1186/1471-2164-11-366. 10.1186/1471-2164-11-366 PMC3017763 20537123

[27] Bhattacharya D, Pelletreau KN, Price DC, Sarver KE, Rumpho ME (2013). Genome analysis of *Elysia chlorotica* egg DNA provides no evidence for horizontal gene transfer into the germ line of this kleptoplastic mollusc. Mol Biol Evol.

[28] Hehenberger E, Gast RJ, Keeling PJ (2019). A kleptoplastidic dinoflagellate and the tipping point between transient and fully integrated plastid endosymbiosis. Proc Natl Acad Sci USA.

[29] Gast RJ, Moran DM, Dennett MR, Caron DA (2007). Kleptoplasty in an Antarctic dinoflagellate: caught in evolutionary transition?. Environ Microbiol.

[30] Gottsching M, Čalasan AŽ, Kretschmann J (2017). Two new generic names for dinophytes harbouring a diatom as an endosymbiont, *Blixaea* and *Unruhdinium* (Kryptoperidiniaceae, Peridiniales). Phytotaxa.

[31] Chesnick JM, Kooistra WHCF, Wellbrock U (1997). Ribosomal RNA analysis indicates a benthic pennate diatom ancestry for the endosymbionts of the dinoflagellates *Peridinium foliaceum* and *Peridinium balticum* (Pyrrhophyta). J Eukaryot Microbiol.

[32] Yamada N, Sym SD, Horiguchi T (2017). Identification of highly divergent diatom-derived chloroplasts in dinoflagellates, including a description of *Durinskia kwazulunatalensis* sp. nov. (Peridiniales, Dinophyceae). Mol Biol Evol.

[33] Yamada N, Bolton JJ, Trobajo R, Mann DG, Dąbek P, Witkowski A, et al. Discovery of a kleptoplastic ‘dinotom’ dinoflagellate and the unique nuclear dynamics of converting kleptoplastids to permanent plastids. Sci Rep. 2019; 10.1038/s41598-019-46852-y. 10.1038/s41598-019-46852-y PMC6642167 31324824

[34] Tippit DH, Pickett-Heaps JD (1976). Apparent amitosis in the binucleate dinoflagellate *Peridinium balticum*. J Cell Sci.

[35] Kempton JW, Wolny J, Tengs T, Rizzo P, Morris R, Tunnell J (2002). *Kryptoperidinium foliaceum* blooms in South Carolina: a multi-analytical approach to identification. Harmful Algae.

[36] Okolodkov YB, Merino-Virgilio FC, Huerta-Quintanilla DA, Gárate-Lizárraga I, Steidinger KA, Aguilar-Trujillo AC (2020). A Kryptoperidiniaceae species (Dinophyceae: Peridiniales) blooming in coastal Yucatan waters, Gulf of Mexico. Protistology.

[37] Tomas RN, Cox ER, Steidinger KA (1973). *Peridinium balticum* (Levander) Lemmermann, an usual dinoflagellate with a mesocaryotic and an eucaryotic nucleus. J Phycol.

[38] Pienaar RN, Sakai H, Horiguchi T (2007). Description of a new dinoflagellate with a diatom endosymbiont, *Durinskia capensis* sp. nov. (Peridiniales, Dinophyceae) from South Africa. J Plant Res.

[39] Yamada N, Sakai H, Onuma R, Horiguchi T. Five non-motile dinotom dinoflagellates of the genus *Dinothrix*. Front Plant Sci. 2020; 10.3389/fpls.2020.591050. 10.3389/fpls.2020.591050 PMC7710806 33329655

[40] Jeffrey SW, Vesk M (1976). Further evidence for a membrane-bound endosymbiont within the dinoflagellate *Peridinium foliaceum*. J Phycol.

[41] Stoecker DK, Johnson MD, de Vargas C, Not F (2009). Acquired phototrophy in aquatic protists. Aquat Micro Ecol.

[42] Mitra A, Flynn KJ, Tillman U, Raven JA, Caron D, Stoecker DK (2016). Defining planktonic protist functional groups on mechanisms for energy and nutrient acquisition: Incorporation of diverse mixotrophic strategies. Protist.

[43] Mann DG, Yamada N, Bolton JJ, Witkowski A, Trobajo R (2023). *Nitzschia captiva* sp. nov., the essential prey diatom of the kleptoplastic dinoflagellate *Durinskia capensis*, compared with *N. agnita*, *N. kuetzingioides* and other species. Phycologia.

[44] Gilbert M, Wilhelm C, Richter M (2000). Bio-optical modelling of oxygen evolution using in vivo fluorescence: Comparison of measured and calculated photosynthesis/irradiance (PI) curves in four representative phytoplankton species. J Plant Physiol.

[45] Horiguchi T, Pienaar RN (1994). Ultrastructure of a new marine sand-dwelling dinoflagellate *Gymnodinium quadrilobatum* sp. nov. (Dinophyceae) with special reference to its endosymbiotic alga. Eur J Phycol.

[46] Mann DG, Trobajo R, Sato S, Li C, Witkowski A, Rimet F, et al. Ripe for reassessment: A synthesis of available molecular data for the speciose diatom family Bacillariaceae. Mol Phylogenet Evol. 2021; 10.1016/j.ympev.2020.106985. 10.1016/j.ympev.2020.106985 33059066

[47] Kashiyama Y, Yokoyama A, Kinoshita Y, Shoji S, Miyashiya H, Shiratori T (2012). Ubiquity and quantitative significance of detoxification catabolism of chlorophyll associated with protistan herbivory. Proc Natl Acad Sci USA.

[48] Allen JF (2003). Cyclic, pseudocyclic and noncyclic photophosphorylation: new links in the chain. Trends Plant Sci.

[49] Ast M, Gruber A, Schmitz-Esser S, Neuhaus HE, Kroth PG, Horn M (2009). Diatom plastids depend on nucleotide import from the cytosol. Proc Natl Acad Sci USA.

[50] Chu L, Gruber A, Ast M, Schmitz-Esser S, Altensell J, Neuhaus HE (2016). Shuttling of (deoxy) purine nucleotides between compartments of the diatom *Phaeodactylum tricornutum*. N Phytol.

[51] Bailleul B, Berne N, Murik O, Petroutsos D, Prihoda J, Tanaka A (2015). Energetic coupling between plastids and mitochondria drives CO_2_ assimilation in diatoms. Nature.

[52] Hehenberger H, Burki F, Kolisko M, Keeling PJ (2016). Functional relationship between a dinoflagellate host and its diatom endosymbiont. Mol Biol Evol.

[53] Rauch C, Tielens AGM, Serôdio J, Gould SB, Christa G. The ability to incorporate functional plastids by the sea slug *Elysia viridis* is governed by its food source. Mar Biol. 2018; 10.1007/s00227-018-3329-8.

[54] Kamikawa R, Tanifuji G, Ishikawa SA, Ishii K, Matsuno Y, Onodera NT (2015). Proposal of a twin arginine translocator system-mediated constraint against loss of ATP synthase genes from nonphotosynthetic plastid genomes. Mol Biol Evol..

[55] Kamikawa R, Moog D, Zauner S, Tanifuji G, Ishida K, Miyashita H (2017). A Non-photosynthetic diatom reveals early steps of reductive evolution in plastids. Mol Biol Evol.

[56] Altenburger A, Cai H, Li Q, Drumm K, Kim M, Zhu Y (2021). Limits to the cellular control of sequestered cryptophyte prey in the marine ciliate *Mesodinium rubrum*. ISME J..

[57] Dodge JD (1969). A review of the fine structure of algal eyespots. Br Phycol J..

[58] Hehenberger E, Imanian B, Burki F, Keeling PJ (2014). Evidence for the retention of two evolutionary distinct plastids in dinoflagellates with diatom endosymbionts. Genome Biol Evol.

[59] Guillard LLL, Ryther JH (1962). Studies on marine planktonic diatoms. I. *Cyclotella nana* Hustedt and *Detonula confervacea* (Cleve) Gran. Can J Microbiol.

[60] Jeffrey SW, Humphrey GF (1975). New spectrophotometric equations for determining chlorophylls *a*, *b*, *c*_1_ and *c*_2_ in higher plants, algae and natural phytoplankton. Biochem Physiol der Pflanz.

[61] Eilers PHC, Peeters JCH (1988). A model for the relationship between light intensity and the rate of photosynthesis in phytoplankton. Ecol Model.

[62] Sprecher BN, Buck JM, Ropella LL, Ramsperger A, Kroth PG, Yamada N. Genetic transformation methods for diatom *Nitzschia captiva*: New tools to better understand dinotom endosymbiosis. Algal Res. 2023; 10.1016/j.algal.2023.103136.

[63] Apt KE, Zaslavkaia L, Lippmeier JC, Lang M, Kilian O, Wetherbee R (2002). In vivo characterization of diatom multipartite plastid targeting signals. J Cell Sci.

[64] Gruber A, Vugrinec S, Hempel F, Gould SB, Maier UG, Kroth PG (2007). Protein targeting into complex diatom plastids: functional characterization of a specific targeting motif. Plant Mol Biol.

[65] Alverson AJ, Jansen RK, Theriot EC (2007). Bridging the Rubicon: Phylogenetic analysis reveals repeated colonizations of marine and fresh waters by thalassiosiroid diatoms. Mol Phylogenet Evol.

[66] Kumar S, Stecher G, Li M, Knyaz C, Tamura K (2018). MEGA X: Molecular evolutionary genetics analysis across computing platforms. Mol Biol Evol.

[67] Pfaffl MW (2001). A new mathematical model for relative quantification in real-time RT-PCR. Nucleic Acids Res.

[68] Coesel S, Mangogna M, Ishikawa T, Heijde M, Rogato A, Finazzi G (2009). Diatom PtCPF1 is a new cryptochrome/photolyase family member with DNA repair and transcription regulation activity. EMBO Rep..

