# Supplementary material for: Prey preference in a kleptoplastic dinoflagellate is linked to photosynthetic performance
Source: ISME J. 2023 Jun 30;17(10):1578–88. doi: 10.1038/s41396-023-01464-3 (PMC10504301; doi:10.1038/s41396-023-01464-3)
Supplement: Supplementary file 1 — Supplementary figures and tables [file 41396_2023_1464_MOESM1_ESM.pdf]

Title: Prey preference in a kleptoplastic dinoflagellate is linked to photosynthetic performance

The name of the authors: Norico Yamada, Bernard Lepetit, David G. Mann, Brittany N. Sprecher, Jochen M. Buck, Paavo Bergmann, Peter G. Kroth, John J. Bolton, Przemysław Dąbek, Andrzej Witkowski, So-Yeon Kim, Rosa Trobajo

Supplementary Figures

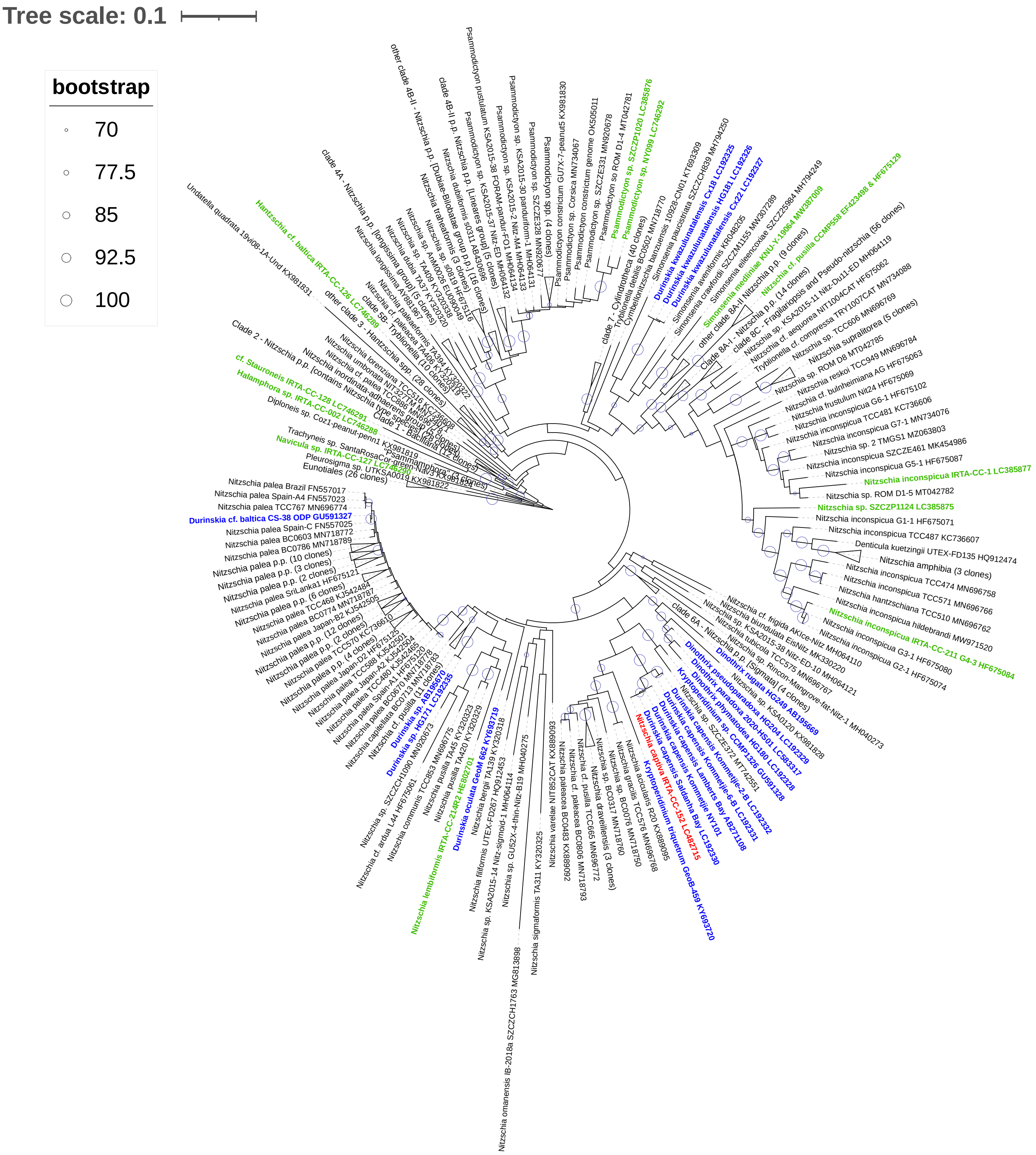

Supplementary Figure 1: Molecular phylogeny of Bacillariaceae, based on a maximum-likelihood analysis of available *rbc*L sequences and rooted with Eunotiales. The Bacillariaceae comprises several apparently monophyletic genera (e.g. *Cylindrotheca*, *Psammodictyon*, *Simonsenia*, *Pseudo-nitzschia*), which are nested within the large paraphyletic genus *Nitzschia* [46], which we refer to here (Supplementary Table 1) as “*Nitzschia sensu lato*”. The type species of *Nitzschia*, *N. sigmoidea*, does not belong to the same clade as the dinotom ODPs. Light green = diatoms used in this study. Blue = ODPs of dinotoms. Red = the essential diatom of *D. capensis*, *N. captiva*. The *D. capensis* (strain NY101) sequence is identical to *N. captiva*'s sequence (LC482715).

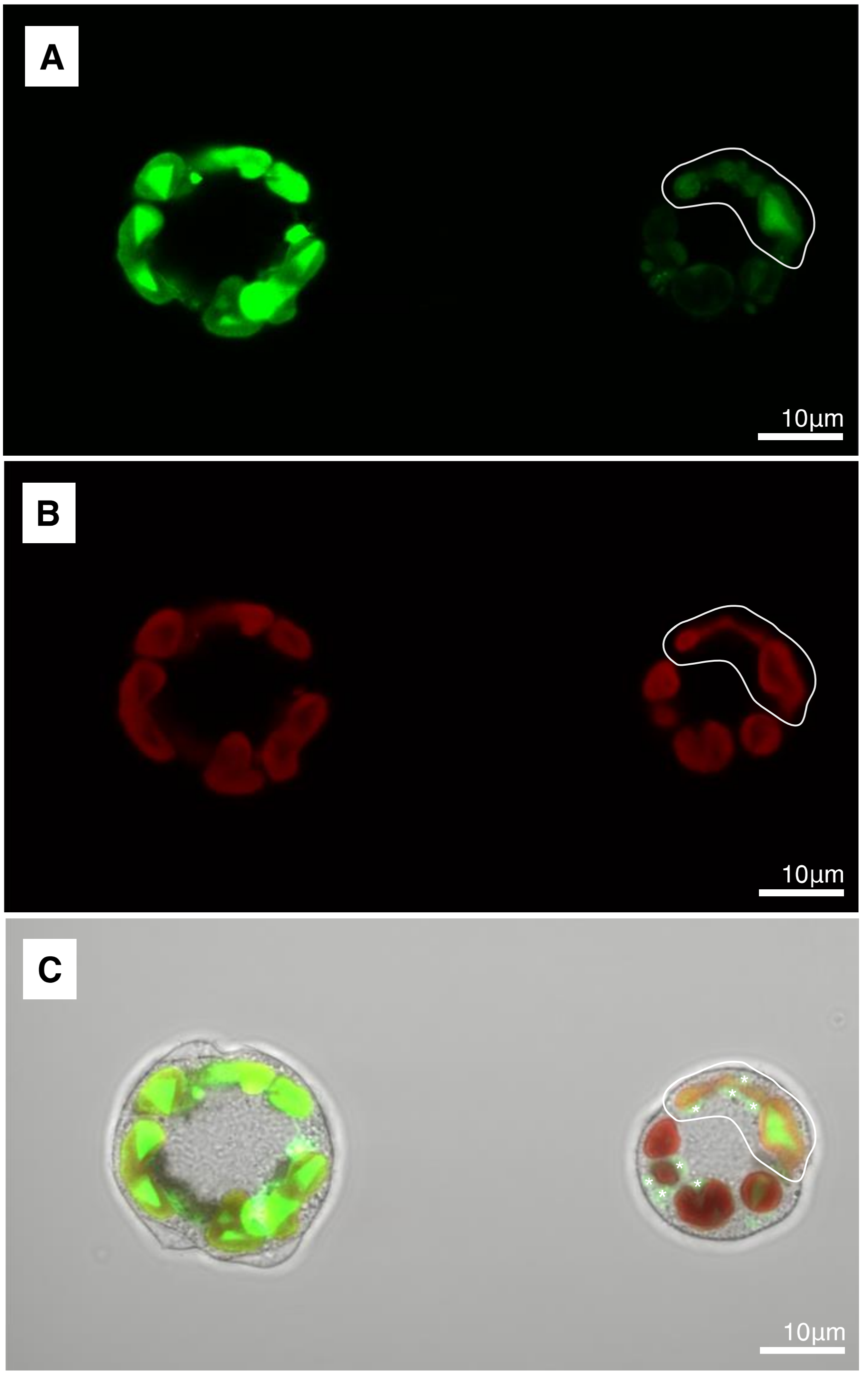

Supplementary Figure 2: *D. capensis* possessing only GFP-expressing *Nitzschia captiva*-derived plastids (left cell) and possessing both GFP-expressing *N. captiva*-derived and wild-type *N. inconspicua*-derived plastids (right cell). **(A)** All plastids of the left cell had GFP fluorescence, while in the right cell, only some of them (white circle) were GFP-expressing *N. captiva*-derived plastids. The rest of plastids in the right cell were wild-type *N. inconspicua* plastids. **(B)** All plastids of the left and right cells had Chl *a* autofluorescence. **(C)** A merged photo of GFP fluorescence, Chl *a* fluorescence and blight field. GFP parts of wild-type *N. inconspicua*-derived compartment (asterisk) in the right cell were most likely from the *N. captiva*-derived cytoplasm.

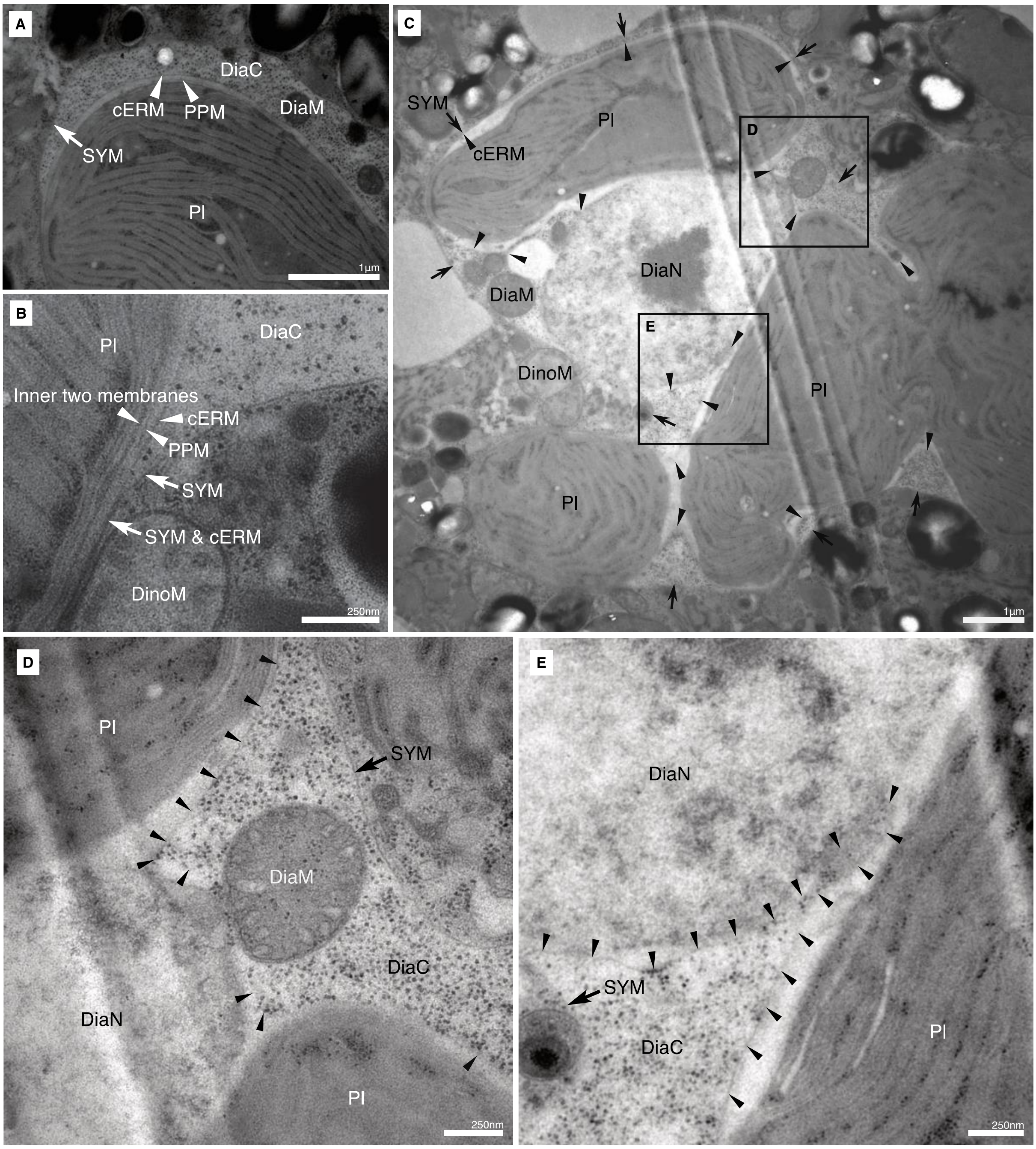

Supplementary Figure 3: *Nitzschia captiva*-derived ODPs of *D. capensis.* **(A and B)** Diatom plastids (Pl) were surrounded by five membranes: the symbiosome membrane (SYM), the outermost plastidial membrane (= chloroplast endoplasmic reticulum membrane; cERM), the second outermost plastidial membrane (= periplastid membrane; PPM), and two inner two membranes of plastids. **(C)** The cERM was continuous with the diatom nuclear envelope membrane. Arrow = SYM. Arrowhead = cERM. **(D and E)** Enlarged photos of C. The arrowheads indicate the cERM and the connected nuclear envelope. DiaC = Diatom cytoplasm, DiaM = Diatom mitochondria, DiaN = Diatom nucleus, DinoM = Dinoflagellate mitochondria.

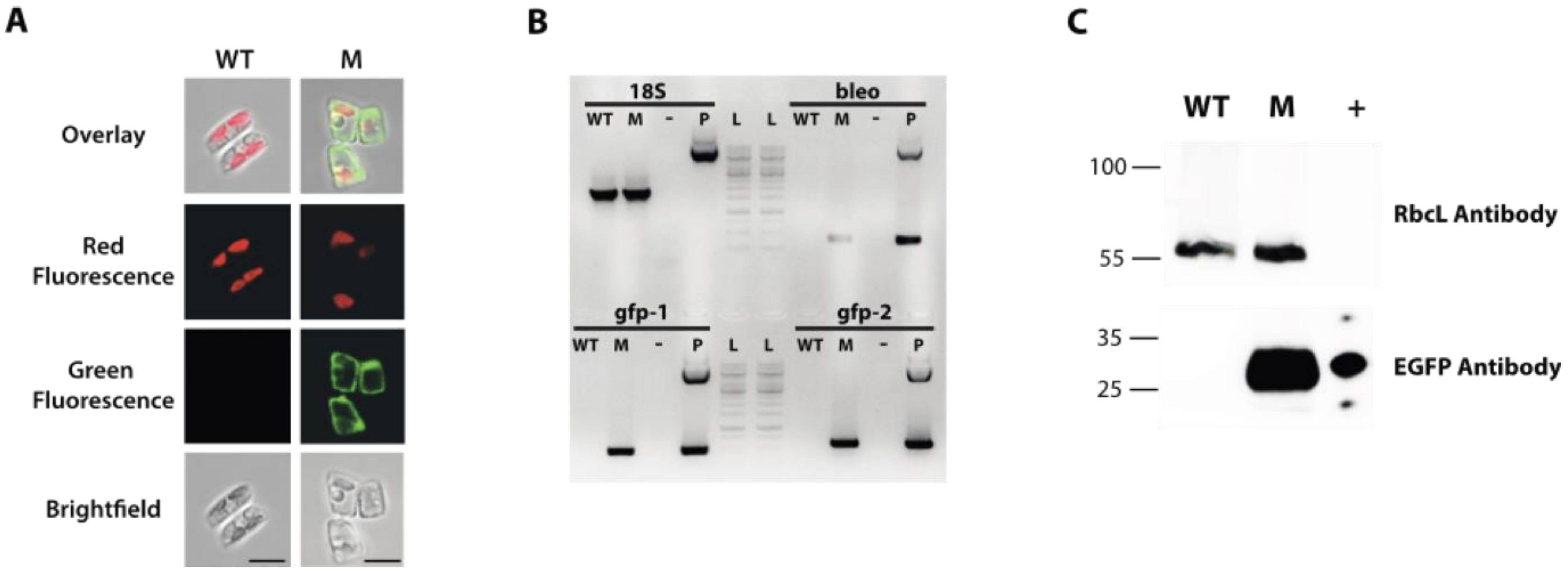

Supplementary Figure 4: Confirmation of GFP expression in *Nitzschia inconspicua*. **(A)** CLSM of *N. inconspicua* cells. Scale bar is 5 µm. GFP expression is seen in the *N. inconspicua* mutant (M) but not in the wild type (WT). **(B)** PCRs using gDNA with four separate primer sets to amplify the 18S ribosomal DNA (18S) as a positive control, and the zeocin resistance gene (bleo) and the EGFP gene (gfp-1, gfp-2) to prove successful vector introduction to *N. inconspicua.* P = plasmid, - = no template added. **(C)** Western Blot for the rbcL and EGFP genes. + = EGFP purified protein.

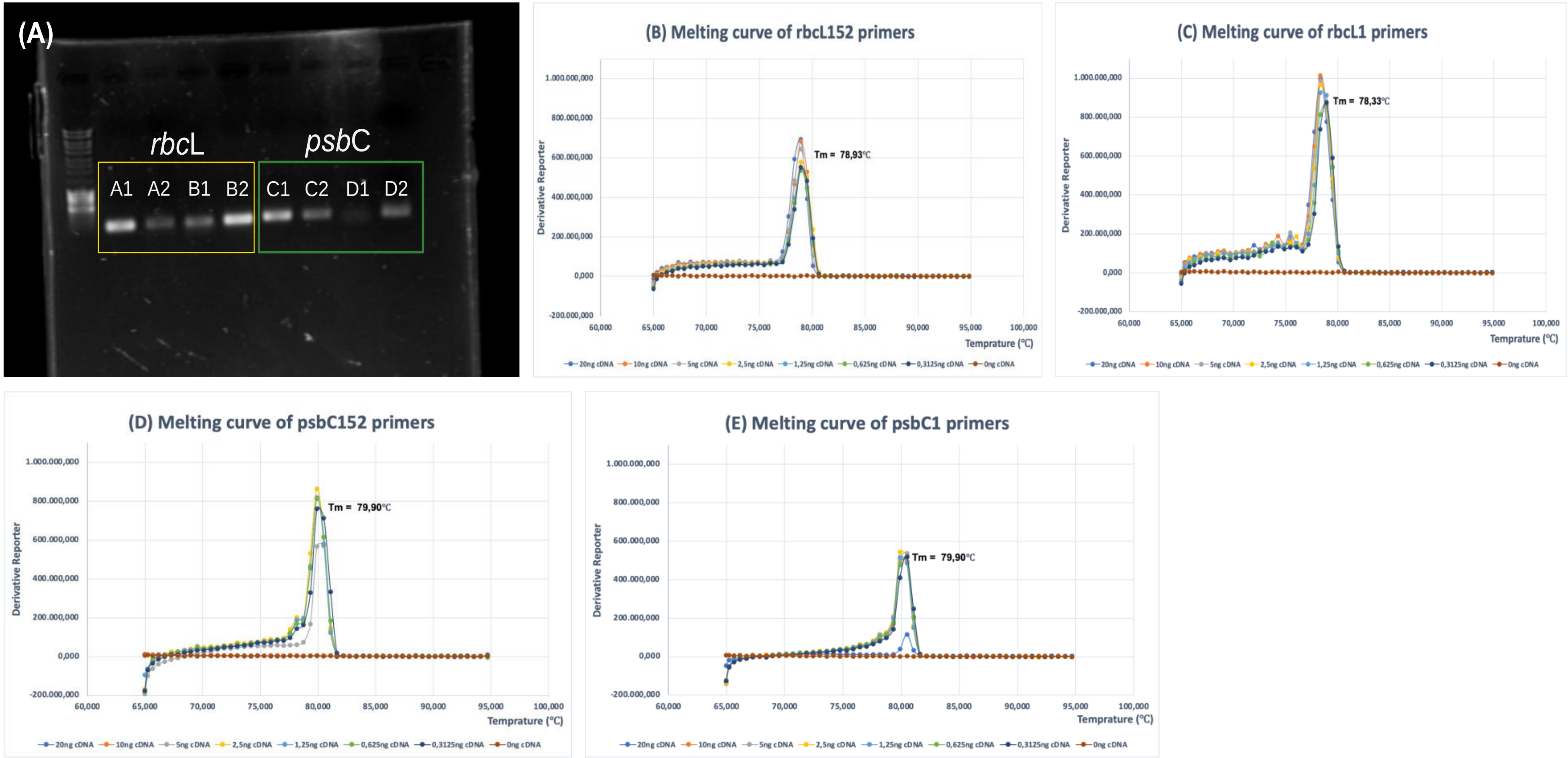

Supplementary Figure 5: RT-qPCR Primer evaluation. **(A)** PCR results using species-selective primers. The primers designed for *N. captiva* or *N. inconspicua* (Supplementary Table 3) were able to amplify the target gene of each diatom species-selectively, although they also amplified non-target genes with low efficiency. A: rbcL152 primers designed for *N. captiva.* B: rbcL1 primers designed for *N. inconspicua.* C: psbC152 primers designed for *N. captiva.* D: psbC1 primers designed for *N. inconspicua.* 1: DNA extracted from free-living *N. captiva*. 2: DNA extracted from free-living *N. inconspicua*.

(B-E) Melting curves of species-selective primers generated via RT-qPCR. **(B)** rbcL152 primers. **(C)** rbcL1 primers. **(D)** psbC152 primers. **(E)** psbC1 primers. Free-living *N. captiva* or free-living *N. inconspicua* were used for rbcL/psbC152 or rbcL/psbC1 primers, respectively. Due to high cDNA concentration, 10 ng and 20 ng of cDNA / 20 µl reaction were undetectable in psbC152 primers, and 20 ng of cDNA / 20 µl reaction had a small peak in psbC1 primers.

Supplementary Tables

Supplementary Table 1: The average cell numbers of *D. capensis* per ml in feeding experiments.

**Species name**	**Strain**	***D. capensis* cells / ml (average of four-bioreplicates)**			
		**Day 0**	**Day 14**	**Day 28**	**Day 42**
Group 1: *Nitzschia sensu lato* collected from the type locality of *D. capensis*					
Hanitzschia cf. *baltica*	IRTA-CC-126	10	765	3616	6838
***Nitzschia captiva* (essential diatom)**	**IRTA-CC-152**	**10**	**853**	**23090**	**76890**
Nitzschia inconspicua	IRTACC-1	10	1210	7833	21195
Psammodictyon sp.	NY099	10	752	10965	18640
Group 2: Diatoms of other genera collected from the type locality of *D. capensis*					
Halamphora sp.	IRTA-CC-2	10	1317	11815	17065
cf. *Stauroneis* sp.	IRTA-CC-128	10	1391	4817	9875
Navicula sp.	IRTA-CC-127	10	880	9548	15193
Group 3: *Nitzschia sensu lato* collected from other places					
Nitzschia inconspicua	IRTA-CC-211	10	1393	20028	28435
Nitzschia lembiformis	IRTA-CC-214	10	1487	9453	10635
Nitzschia cf. *pusilla*	CCMP558	10	825	1598	1670
Nitzschia sp.	SZCZP1124	10	784	6885	14623
Psammodictyon sp.	SZCZP1020	10	1524	14140	25304
Simonsenia medliniae	KNU-Y-19064	10	615	443	653
Controls					
No diatom	-	10	433	743	1238
Mixing of three diatoms	IRTA-CC-152<br>IRTACC-1<br>NY099	10	4849	99253	136357

Supplementary Table 2: Free-living diatom contamination rate for each sample.

Sample name	Sample description	Experiment	Free-living diatom contamination rate (%, average of three bio-replicates)
c Day 0	*D. capensis* cultured with *N. captiva* Day 0	PAM/Chl *a*	0
d Day 0	*D. capensis* cultured with *N. captiva* + *N. inconspicua* Day 0		0
e Day 0	*D. capensis* cultured with *N. inconspicua* Day 0		47.21 (due to the highly residue free-living *N. inconspicua*, sample e was not used for experiments)
d Day 0	*D. capensis* cultured with *N. captiva* + *N. inconspicua* Day 0	RT-qPCR	2.87
d S-Day 3	*D. capensis* cultured with *N. captiva* + *N. inconspicua* S-Day 3		1.20
d S-Day 5	*D. capensis* cultured with *N. captiva* + *N. inconspicua* S-Day 5		0
d S-Day 7	*D. capensis* cultured with *N. captiva* + *N. inconspicua* S-Day 7		0
d S-Day 10	*D. capensis* cultured with *N. captiva* + *N. inconspicua* S-Day 10		0
d S-Day 14	*D. capensis* cultured with *N. captiva* + *N. inconspicua* S-Day 14		0
c Day 0	*D. capensis* cultured with *N. captiva* Day 0	Qphar	6.04
d Day 0	*D. capensis* cultured with *N. captiva* + *N. inconspicua* Day 0		4.71
c Day 0	*D. capensis* cultured with *N. captiva* Day 0	Western blot	2.65

Supplementary Table 3: The primer list and the amplicon sequences of *N. inconspicua* with RT-qPCR.

Primer	Sequence 5' to 3	Target species	Target gene	The sequences of RT-qPCR amplicons of *N. inconspicua* at Day 0
psbC22	CGTGGTGATACATAGTTA	Diatoms	psbC	
psbC1154	GCACACGCTGGNTTAATGG	Diatoms	psbC	
rbcL152-F	AACGCGGCTAAGACATGTGG	*N. captiva*	rbcL	
rbcL152-R	CTGTTGGTGTTTCAGCGAAA	*N. captiva*	rbcL	
rbcL1-F	AACGCTGCGAAAACATGTGG	*N. inconspicua*	rbcL	No amplicon
rbcL1-R	CTGTTGGTGTTGCAGCGAAA	*N. inconspicua*	rbcL	No amplicon
psbC152-F	GTCGCGCATTTGTTTGGTCT	*N. captiva*	psbC	
psbC152-R	AGATGCTTCCGGACCAGTTG	*N. captiva*	psbC	
psbC1-F	GTCGTGCTTTTGTATGGTCA	*N. inconspicua*	psbC	TATTTCTTTATGGGATTCACAGCATCATT ATATTCTTGGTATAATAACACTGCATATC CTAGTGAATTATACGGACCAACAGGTCC AGAAGCATCAAGGG
psbC1-R	TGATGCTTCTGGACCTGTTG	*N. inconspicua*	psbC	TTATTATACCAAGAATATAATGATGCTGT GAATCCCATTAAAGAAATAGCAGCTAAA CTGTATGAAAGGTATGCTTCACCTGACC ATACAAAAGCACGACC
H18S-F	GACCTATCAGCTTCCGACGG	*D. capensis*	18S gene	
H18S-R	TTGCAAGACATGGATGCCCT	*D. capensis*	18S gene	

Supplementary Video 1: A feeding attack of *D. capensis*. After *D. capensis* finds diatoms, it starts attacking them for 5 to 30 minutes, before starting to ingest diatom organelles with its peduncle. Due to the the maximum recording time of our microscope camera (Zeiss Axiocam 305 color, Swiss), each video was recorded for a maximum of 15 mins.

Supplementary Video 2: A single cell of *N. captiva* (arrow) added into a *D. capensis* culture that had been mono-cultured for three weeks in the absence of free-living diatoms. *D. capensis* starts attacking this diatom within 6 minutes. The experiments were repeated 5 times.

Supplementary Video 3: A single cell of *N. inconspicua* (arrow) added into a *D. capensis* culture that had been mono-cultured for three weeks in the absence of free-living diatoms. *D. capensis* never showed any feeding attacks on this diatom during 3 hours. The experiments were repeated 5 times.

Supplementary Video 4: A cell clump of *N. inconspicua* (circle) added into a *D. capensis* culture that had been mono-cultured for three weeks in the absence of free-living diatoms. Contrary to supplementary video 3, *D. capensis* comes close to *N. inconspicua* cells, but after several attacks, it leaves from *N. inconspicua* cells without feeding. The experiments were repeated 5 times.
